# Survey of endoparasites in *Chelonoidis carbonarius* and *Chelonoidis denticulatus* kept at the zoo of the Jungle Warfare Training Center (CIGS), Central Amazon

**DOI:** 10.1590/S1984-29612026041

**Published:** 2026-09-25

**Authors:** Elen Vinhote da Cunha, Dandara de Moraes Geissler, Luciene Almeida Siqueira, Eduardo Lima de Sousa, Adriano Teixeira de Oliveira

**Affiliations:** 1 Instituto Federal do Amazonas – IFAM, Campus Manaus Zona Leste, Manaus, AM, Brasil; 2 Instituto Federal de Educação, Ciência e Tecnologia do Amazonas – IFAM, Núcleo de Estudos de Invertebrados e Vertebrados da Amazônia – NEIVA, Campus Manaus Centro, Manaus, AM, Brasil; 3 Zoológico do Centro de Instrução de Guerra na Selva – CIGS, Manaus, AM, Brasil; 4 Fundação Osvaldo Cruz – FIOCRUZ, Programa de Pós-graduação em Biologia da Interação Patógeno-Hospedeiro – PPGBIO-Interação, Manaus, AM, Brasil; 5 Instituto Federal de Educação, Ciência e Tecnologia do Amazonas – IFAM, Centro Universitário Nilton Lins – CUNL, Programa de Pós-graduação em Ciências Veterinárias – PPGCVET, Manaus, AM, Brasil; 6 Universidade Federal do Amazonas – UFAM, Programa de Pós-graduação em Ciência Animal e Recursos Pesqueiros – PPGCARP, Manaus, AM, Brasil

**Keywords:** Coproparasitological, chelonians, gastrointestinal parasites, public health, Coproparasitologia, quelônios, parasitas gastrointestinais, saúde pública

## Abstract

The genus *Chelonoidis* tortoises are terrestrial reptiles susceptible to gastrointestinal endoparasites, mainly due to improper management. The present study aims to analyze the frequency of gastrointestinal parasites detected in samples from *Chelonoidis carbonarius* and *Chelonoidis denticulatus*, and to identify and characterize taxonomically the main parasite types. Seven samples of fresh feces were collected from enclosures at the Jungle Warfare Training Center (CIGS) Zoo in Manaus, Amazonas, and evaluated. A high frequency of intestinal endoparasites was observed, with nematode eggs from the families Ascarididae (suggestive of *Angusticaecum* spp.) (35.5%), Oxyurida (6.3%), and Strongylidae/Ancylostomatidae (strongylid type) (25%) being identified. Among the protozoa, oocysts of *Eimeria* spp. (6.3%), *Cystoisospora* spp. (6.3%), and *Cyclospora* spp. (6.3%) were detected, in addition to the trophozoite of Ciliophora (6.3%). It has been found that captive animals are prone to helminth and protozoan parasitic infections due to limited space and contact with waste, making it necessary to encourage coproparasitological analysis and studies in reptiles to ensure knowledge of diseases related to gastrointestinal parasites and promote control, prophylaxis, and treatment measures that minimize possible risks to animal and public health.

## Resumo

Os jabutis do gênero *Chelonoidis* são répteis terrestres suscetíveis a endoparasitos gastrointestinais, principalmente devido ao manejo inadequado. O presente estudo tem como objetivo analisar a frequência de formas parasitárias gastrointestinais detectadas em amostras de *Chelonoidis carbonarius* e *Chelonoidis denticulatus*, a fim de identificar e caracterizar taxonomicamente os principais tipos de parasitos. Foram avaliadas sete amostras de fezes frescas, coletadas em recintos do Zoológico do Centro de Instrução de Guerra na Selva (CIGS), em Manaus, Amazonas. Observou-se alta frequência de endoparasitos intestinais, com identificação de ovos de nematoides da família Ascarididae: (sugestivos de *Angusticaecum* spp.). (35,5%), Oxyurida (6,3%) e Strongylidae/Ancylostomatidae (tipo strongídeos) (25%). Entre os protozoários, foram detectados oocistos de *Eimeria* spp. (6,3%), *Cystoisospora* spp. (6,3%) e *Cyclospora* spp. (6,3%), além de formas trofozoíticas de Ciliophora (6,3%). Verificou-se que os animais em cativeiro são propensos a infecções parasitárias por helmintos e protozoários devido ao espaço limitado e ao contato com dejetos, o que torna necessárias análises e estudos coproparasitológicos em répteis para garantir o conhecimento das doenças relacionadas a parasitos gastrointestinais e promover medidas de controle, profilaxia e tratamento que minimizem os riscos à saúde animal e à saúde pública.

Endoparasites are organisms that live and develop inside a host, such as protozoa and helminths (nematodes, cestodes, and trematodes) (Arenas, 2020). Endoparasites generally have tropism for regions such as the gastrointestinal tract, blood, and lungs (Taylor et al., 2017). In reptilian veterinary medicine, Bardi et al. (2019) reported that parasitic diseases are the most common causes of disease and death in reptiles. Thus, understanding the relationship between reptiles and their parasites is established in two ways: through the installation of the parasite in immunocompetent animals or through external factors that cause an imbalance between the animal and the environment (overcrowding, poor hygiene, inadequate environmental and food management, concomitant diseases, among others) that can reduce the immune response of the hosts and promote the persistence and pathogenicity of the parasites (Bardi et al., 2019). Thus, the pathological state depends largely on changes in the host-parasite-environment relationship (Forattini, 2001).

The growing demand for exotic animals as unconventional pets has increased the need for specialized care in veterinary clinics (Marques et al., 2020). In addition, captive breeding and illegal trade, whether for food consumption or for other derivatives, are among the main reasons to study these parasites in reptiles, as reptiles harbor a wide variety of pathogens that can affect humans (Garcês et al., 2026). It is also important to note that these parasites can cause diseases that are not apparent in animals but cause serious health problems in humans (Rataj et al., 2011).

A study conducted at the Slovenian Zoo reported relevant findings of endoparasites in chelonians, including oxyurid nematodes such as Pharyngodonidae, *Tachygonetria* spp., strongylid nematodes such as *Camallanus* spp., and *Balantioides* spp. and trematode eggs were also found; *Strongyloides* spp., Cestoda, and *Nyctotherus* spp. were less prevalent (Rataj et al., 2011).

In another study conducted in Germany, five metazoan species (class Nematoda) were found in fecal samples from 19 turtle species, among which oxyurids were the most common, namely *Angusticaecum* spp., *Strongyloides* spp., and *Heterakis* spp. (Hallinger et al., 2018). In addition, a diversity of protozoa such as *Hexamita* spp., *Tritrichomonas* spp, facultative flagellates such as *Hexamatrix* spp. and *Trimituss* spp., two different genera of amoebae (*Hartmanella* spp. and *Entamoeba* spp.), a genus of ciliates, *Balantioides* spp., and yeasts such as *Blastocystis* spp. were also found in the fecal samples (Hallinger et al., 2018). The occurrence of endoparasites is also high among reptiles raised in captivity, but research and coproparasitological evaluations are scarce in Brazilian zoos. In a study conducted at the Matinha Municipal Park Zoo, Itapetinga, Bahia, several taxa of gastrointestinal parasites were identified in the form of cysts, oocysts, and eggs, including 8 protozoa and 19 helminths, with a predominance of coccidia, Oxyurideae, and *Angusticaecum* spp. for reptiles (Silva et al., 2022).

Tortoises are robust terrestrial animals with compact bodies and cylindrical limbs, well-adapted to rugged environments. The predominant genus in Brazil is *Chelonoidis*: *C. carbonarius* (red-footed tortoise) and *C. denticulatus* (yellow-footed tortoise), which are widely distributed, and *C. chilensis*, which is rarer and only occurs in the extreme south of the country (Cubas & Baptistotte, 2007). The species *C. carbonarius* occurs from Central America to South America. *C. denticulatus* occurs in the northern half of South America, extending to southern Brazil and covering the Andes (Freire et al., 2017).

Meireles et al. (2018) found that animals living in captivity are more prone to parasites than those living in the wild, due to environmental factors, hygiene, and enclosure management. In a joint breeding of *C. carbonarius* and *C. denticulatus*, fecal samples were collected and examined using different techniques. The findings include eggs and cysts of various parasites, with *Strongyloides* spp. being the most frequent, followed by nematode eggs from the family Strongylididae.

The study of endoparasites in *C. carbonarius* and *C. denticulatus* is essential for understanding their frequency in these hosts and their potential harmful effects on animal and human health. Research already conducted indicates that these animals have great potential to be reservoirs because they carry a high diversity of parasites, which reinforces the need for attention and care to health, as well as attention to good health surveillance practices and potential zoonotic risk because they are directly linked to public health (Rataj et al., 2011). The present study aims to analyze the frequency of gastrointestinal parasitic forms detected in the studied samples from *C. denticulatus* and *C. carbonarius* tortoises housed at the Zoo of the Jungle Warfare Training Center (CIGS), Manaus, Amazonas, Brazil.

The study was conducted at the CIGS zoo, located in Manaus, State of Amazonas, Brazil (Figure 1). The target population consisted of captive tortoises of the species *C. carbonarius* and *C. denticulatus* (Figure 2). The animals are housed in two specific enclosures for each species. Each enclosure is an open environment, delimited by a small wall, with a predominantly sandy substrate, containing few shelters, and located close to the visitor area (Supplementary 1). Each of these environments houses groups of approximately 20 tortoises, where they reproduce freely. For this survey, the selection of animals was random, considering that the individuals were adults and originated from these collective enclosures. Thus, the final sample consisted of three adult individuals of *C. carbonarius* and four of *C. denticulatus*, for a total of seven animals.

**Figure 1:**
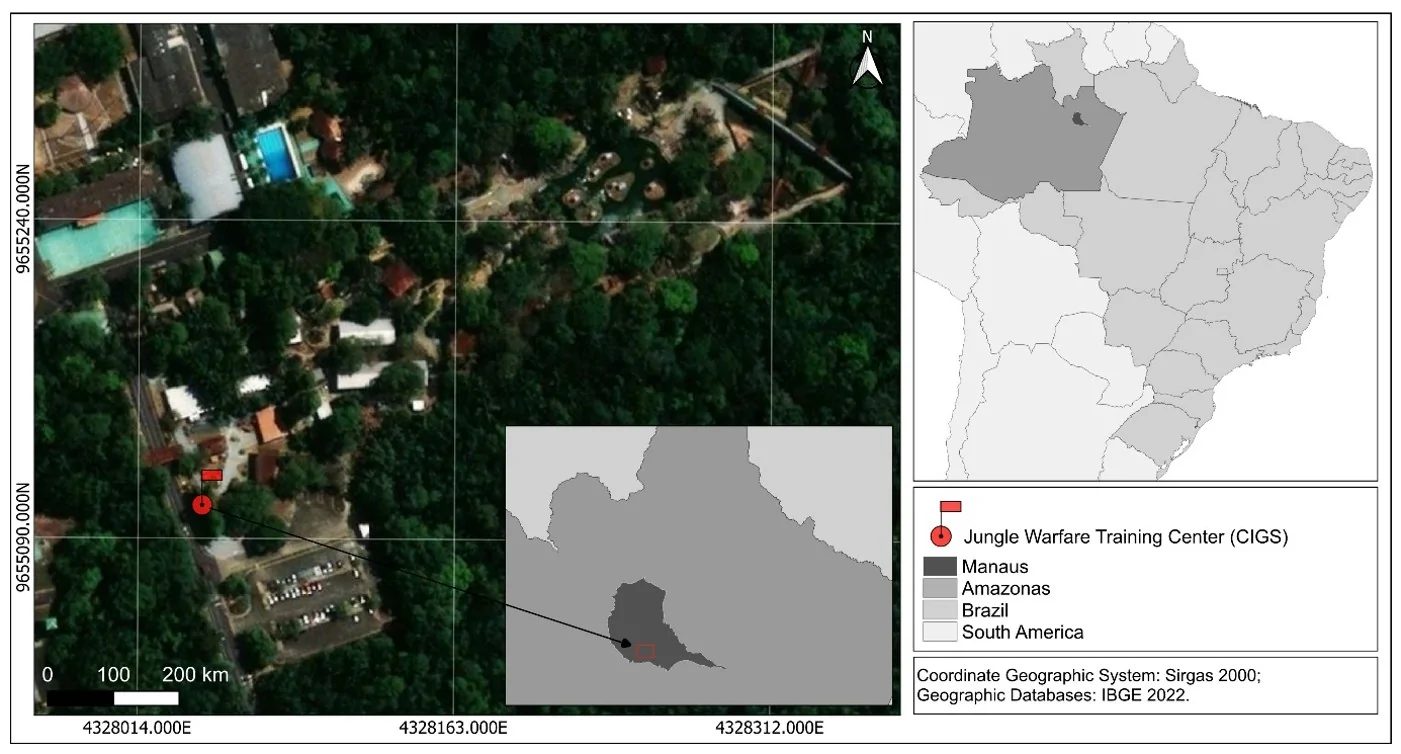
Location map of the Jungle Warfare Training Center (CIGS) Zoo in Manaus, Amazonas.

**Figure 2:**
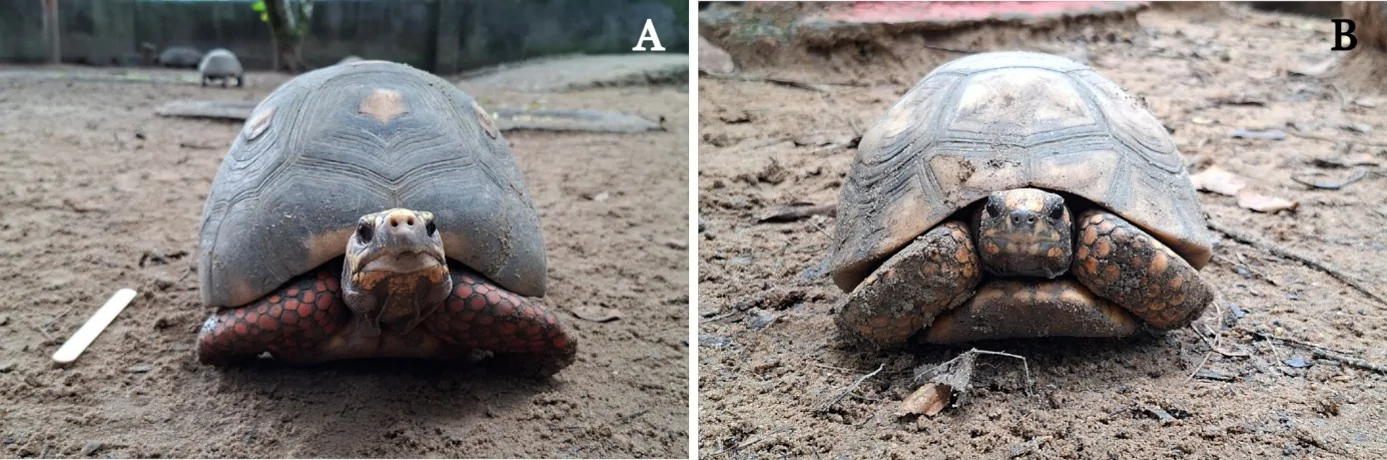
Chelonoidis tortoise species. (A) *Chelonoidis carbonarius* (red-footed tortoise) and (B) *Chelonoidis denticulatus* (yellow-footed tortoise) from the CIGS Zoo, Manaus, Amazonas, Brazil.

Fecal samples were collected in the morning (between 9:00 a.m. and 10:00 a.m.). For most animals (n=6), collection was performed on two consecutive days (August 24 and 25, 2025) in clean environments to which they were temporarily transferred, ensuring the known origin of the material. At the same time, samples from a previously isolated juvenile *C. denticulatus* (clinical case) were collected on different dates (August 9 and 12, 2025), directly from its individual containment enclosure. All samples (n=7) were individually labeled and stored in correctly labeled sterile jars. All samples were processed at the Multidisciplinary Laboratory of the Federal Institute of Amazonas Campus Manaus East Zone (IFAM-CMZL) and Center for the Study of Invertebrates and Vertebrates of the Amazon (NEIVA) of the Federal Institute of Amazonas Campus Manaus East Zone (IFAM-CMC).

Initially, a direct fecal smear was performed. In this technique, a small portion of feces (less than 1 g) was homogenized with one to two drops of distilled water between a slide and a coverslip for fresh examination, allowing for initial research into parasitic structures. At the same time, the centrifugal flotation method (Willis-Mollay method) was performed. For this procedure, approximately 2g of feces were homogenized with 20 mL of saturated saline solution. This mixture was sieved, and the filtered liquid was transferred to a 10 mL test tube, filling it to the meniscus. A slide was carefully placed in contact with the meniscus and left to rest for 20 minutes. After this period, the slide was collected for analysis, aiming to recover light eggs and oocysts.

Finally, the spontaneous sedimentation method (Hoffmann, Pons, and Janer method) was used. In this technique, 5g of feces were mixed with distilled water and homogenized. The mixture was sieved and subsequently transferred to a Falcon tube, where it was left to settle for approximately 24 hours. After this period, the supernatant was carefully discarded, and a pipette was used to collect a sediment sample and deposit it on a slide to optimize detection of heavy eggs.

For all preparations obtained by the three methods, the material analyzed on each slide was stained with a drop of Lugol's solution and covered with a coverslip. All slides were examined under an optical microscope (Nikon, Eclipse^®^) with 40x and 100x objective lenses. Parasites were identified based on the morphology of eggs, cysts, and oocysts.

Coproparasitological analysis of feces of *C. carbonarius* and *C. denticulatus* tortoises revealed a high frequency of intestinal endoparasites. Eggs of nematodes from the families Ascarididae (suggestive of *Angusticaecum* spp.), Oxyurida, and Strongylidae/Ancylostomatidae (strongylid type) were identified. Among the protozoa, oocysts of *Eimeria* spp. were sporulated, *Cystoisospora* spp. were sporulated, and *Cyclospora* spp. were detected, in addition to the trophozoites of Ciliophora. The tabulated data also confirms the presence of multiparasitism in samples A1, A2, A3, A4, and A7, highlighting relevant data, especially for juveniles (Table 1).

**Table 1:** Coproparasitological results for *Chelonoidis carbonarius* (red-footed tortoise) and *Chelonoidis denticulatus* (yellow-footed tortoise) from the CIGS zoo, Manaus, Amazonas, Brazil.

**SPECIES**	**SAMPLE ID**	**AGE GROUP**	**RESULT**	**HELMINTHS**	**PROTOZOA**
*C. denticulatus*	A1 and A2	Juvenile	Positive	Ascarididae, Oxyurida	*Eimeria* spp.*, Cystoisospora* spp.*, Cyclospora* spp.
*C. carbonarius*	A3	Adult	Positive	Strongylididae type, Ascarididae	Ciliophora
*C. denticulatus*	A4	Adult	Positive	Strongylididae type, Ascarididae	
*C. carbonarius*	A5	Adult	Negative		
*C. denticulatus*	A6	Adult	Negative		
*C. denticulatus*	A7	Juvenile	Positive	Strongylididae type	Ciliophora*, Entamoeba coli*

Helminths were identified by eggs characteristic of their respective families, and protozoa by cysts found.

The findings show that the Ascarididae (37.5%) and Strongylididae /Ancylostomatidae (strongyle type) (25%) families had the highest frequency rates, mainly due to multiparasitism, with the remaining families accounting for 6.3% of the samples analyzed. This highlights the importance of further studies on reptiles kept in captivity, since many can be asymptomatic carriers, and encourages improvements in and attention to enclosure management and hygiene, emphasizing their zoonotic potential and health risks to animals (Supplementary 2). The methods used were direct smear, flotation, and sedimentation (Taylor et al., 2017).

Under microscopy, nematode eggs were identified as *Angusticaecum* spp. in both species, characterized by their oval shape, thick shells, and smooth surfaces. In *C. carbonarius*, strongyle-type eggs were detected, evidenced by their oval shape, thin and soft shell, and the presence of visible embryonic content. Among the protozoa found in *C. denticulatus*, oocysts of *Eimeria* spp. can be observed, which have an oval shape and a double wall. In contrast, those of *Cyclospora* spp. are spherical and smaller. It was also possible to visualize colorless oocysts of *Cystoisospora* spp., which were less frequently observed in the results and had well-defined contours. Furthermore, in fecal samples from *C. carbonarius*, trophozoites of Ciliophora were identified by their oval shape and granular cytoplasm (Supplementary 3 and 4).

The results obtained in this study reinforce the vulnerability of the species *C. carbonarius* and *C. denticulatus* to various parasites. This frequency can be explained by the environmental conditions (environment) in which they live (Anselmo et al., 2025, 2026; Dias et al., 2025, 2026), corroborating previous studies and reinforcing the need for continuous parasitological monitoring (Freire et al., 2017).

The high frequency of gastrointestinal endoparasites observed in this study is consistent with previous findings in captive *C. carbonarius* and *C. denticulatus* (Silva et al., 2021; Meireles et al., 2018; Rodrigues et al., 2016; Nonnis et al., 2024), with this high parasitic frequency associated with conditions typical of controlled environments, such as high population density in confined spaces and possible deficiencies in hygiene practices, which enhance the transmission and persistence of parasitic cycles (Nonnis et al., 2024).

The present study observed a high parasite load and diversity in the analyzed juvenile *C. denticulatus*, approximately 5 years old. This finding contrasts with trends reported in some of the literature, which often document a higher prevalence and intensity of parasitic infections in adult tortoises (Nonnis et al., 2024; Chávarri et al., 2012). Other ecological and behavioral factors can explain the apparent contradiction observed in the young animal in this study. Gagno (2005) suggests that differences in feeding habits between age groups may directly influence the infection profile. Young tortoises, which may exhibit coprophagy and a more carnivorous or omnivorous diet than essentially herbivorous adults, would be subject to different routes and opportunities for infection. Thus, the high parasite load in juveniles may reflect their initial immune susceptibility, combined with higher-risk behaviors in a captive environment that facilitates contamination. This specific condition may have overlapped with the general tendency for parasites to accumulate with age, highlighting the multifactorial nature of parasite dynamics.

The predominance of strongylid eggs, one of the most frequent findings in this study, was also reported by Meireles et al. (2018), who identified the family Strongylidae as the most prevalent helminth in both tortoise species. Similar results were obtained by Silva et al. (2021), who identified strongylids as the second-most frequent group in both species. However, unlike these authors, no nematode larvae were detected in the analyzed samples; only eggs were found. This high rate can be explained by the ease of transmission, driven by factors such as animal crowding in small areas, which causes stress and reduces animals' resistance to disease, as well as inadequate management conditions, including access to adequate, high-quality food (Freire et al., 2017).

Regarding the described ascarids, the egg morphology suggests the genus *Angusticaecum,* which is commonly reported as parasitizing chelonians (Taylor et al., 2017). This finding is consistent with the study by Silva et al. (2022), which reported a high nematode index for *C. carbonarius*, highlighting the frequency of this genus in these hosts. In addition, studies by Chávarri et al. (2012) found that ascarids were generally more prevalent in captive animals than in free-living animals, supporting the present findings.

The oxyurid eggs found in the samples were reported as prevalent (74.4%) in the study by Nonnis et al. (2024), who performed coproparasitological analyses on feces from turtles of the species *Testudo hermanni*, *T. marginata*, *T. kleinmanni*, *T. graeca*, *Centrochelys sulcata*, *Pyxis arachnoides, Stigmochelys pardalis*, and *Homopus areolatus*, both free-living and captive, with the majority being captive animals. According to Taylor et al. (2017), these nematodes are frequently part of the commensal intestinal fauna of reptiles, a phenomenon also observed by other authors (Rom et al., 2018; Rataj et al., 2011).

Among the protozoa identified, coccidia (*Eimeria* spp., *Cystoisospora* spp., and *Cyclospora* spp.) are considered the most clinically relevant due to their recognized pathogenic potential. Severe coccidiosis may cause significant morbidity and mortality in turtles, particularly when associated with secondary bacterial infections (Garner et al., 2006), highlighting the importance of these parasites in health assessments of captive reptiles. Flagellated protozoa were also detected; however, their presence should be interpreted with caution, as low numbers are commonly reported in the normal intestinal microbiota of healthy reptiles (Springer et al., 2020). Similarly, ciliates were observed, but their taxonomic identity could not be determined beyond the phylum Ciliophora based solely on light microscopy. Although *B. coli* has been reported in reptiles, other ciliates, particularly *Nyctotherus* spp., are frequently found in chelonians and may also constitute components of the normal intestinal fauna. Given the morphological similarity among intestinal ciliates, species-level identification requires complementary morphometric and molecular analyses. Therefore, the detection of Ciliophora should be interpreted conservatively, and its clinical relevance should be evaluated in conjunction with host health status and epidemiological factors.

The occurrence of cysts morphologically compatible with *Entamoeba coli* observed in this study is supported by previous reports of the protozoan in chelonians. *E. coli* has a thin, flexible plasma membrane, granular, vacuolated cytoplasm and a nucleus with irregular peripheral chromatin and an eccentric karyosome. Cysts of the genus *Entamoeba* have been reported in *Chelonia mydas* and *Caretta caretta* by Barata et al. (2024), as well as in *Testudo hermanni* (Wolf et al., 2014). Additionally, Hallinger et al. (2018) reported this genus in *T. hermanni* and *C. carbonarius*, with one cyst per individual detected in positive animals.

The absence of apparent clinical signs in most animals in this study, despite the diverse parasite load, suggests a host-parasite balance. Taylor et al. (2017) state that reptiles kept under appropriate management conditions can remain healthy even when harboring a variety of endoparasites. This observation is corroborated by Meireles et al. (2018), who, despite recording endoparasites in 25 of the 27 samples analyzed, did not observe manifestations of parasitic disease in the animals, indicating that the presence of the parasite does not necessarily imply pathology.

Overall, the results obtained reinforce the vulnerability of *C. carbonarius* and *C. denticulatus* to infections by various endoparasites in captivity. The high frequency observed is directly associated with environmental conditions, such as high population density and poor sanitation, which favor the transmission and maintenance of parasitic cycles, as reported in previous studies.

The study highlighted the high frequency and diversity of gastrointestinal parasites (helminths and protozoa) present in *C. carbonarius* and *C. denticulatus* kept in captivity at the Jungle Warfare Training Center Zoo (CIGS) in Manaus, Amazonas. These findings reinforce the importance of improving care, management, and hygiene in the enclosures, as well as addressing overcrowding. The absence of apparent clinical signs suggests a balance in the host-parasite relationship, but does not exclude risks to animal and public health. Therefore, continuous coproparasitological analysis and monitoring are essential for the effective implementation of management, prophylaxis, and treatment measures for the animals in the herd, to prevent infections and maintain animal welfare.

## Data Availability

Data will be provided to authors upon request.

## Supplementary Material

Supplementary material accompanies this paper.

Supplementary 1 View of the tortoise maintenance enclosures at the CIGS Zoo, Manaus, Amazonas, Brazil.

Supplementary 2 Frequency (%) of endoparasites found in *Chelonoidis carbonarius* (red-footed tortoise) and *Chelonoidis denticulatus* (yellow-footed tortoise) at the CIGS zoo, Manaus, Amazonas, Brazil.

Supplementary 3 Endoparasites are observed in fecal samples from *Chelonoidis denticulatus*. (A) Microscopic field showing: (i) egg suggestive of *Angusticaecum* spp., (ii) oocyst of *Eimeria* spp., (iii) oocyst of *Cyclospora* spp. (B) egg suggestive of *Angusticaecum* spp. (C) oocyst of *Eimeria* spp. (D) oocyst of *Cystoisospora* spp. (E) Microscopic field showing: (iv) cyst of *Cyclospora* spp., (v) oocyst of *Eimeria* spp. (F) oocyst of Cyclospora spp.

Supplementary 4 Endoparasites observed in fecal samples from *Chelonoidis carbonarius*. (A) Ciliophora trophozoite (40x magnification). (B) Strongyle type egg (100x magnification).

This material is available as part of the online article from https://doi.org/10.1590/S1984-29612026041
