# Supplementary material for: Survey of endoparasites in Chelonoidis carbonarius and Chelonoidis denticulatus kept at the zoo of the Jungle Warfare Training Center (CIGS), Central Amazon
Source: Rev Bras Parasitol Vet. 2026 Sep 25;35(3):e013826. doi: 10.1590/S1984-29612026041 (PMC13671223; doi:10.1590/S1984-29612026041)

Supplementary 1. View of the tortoise maintenance enclosures at the CIGS Zoo, Manaus, Amazonas, Brazil.

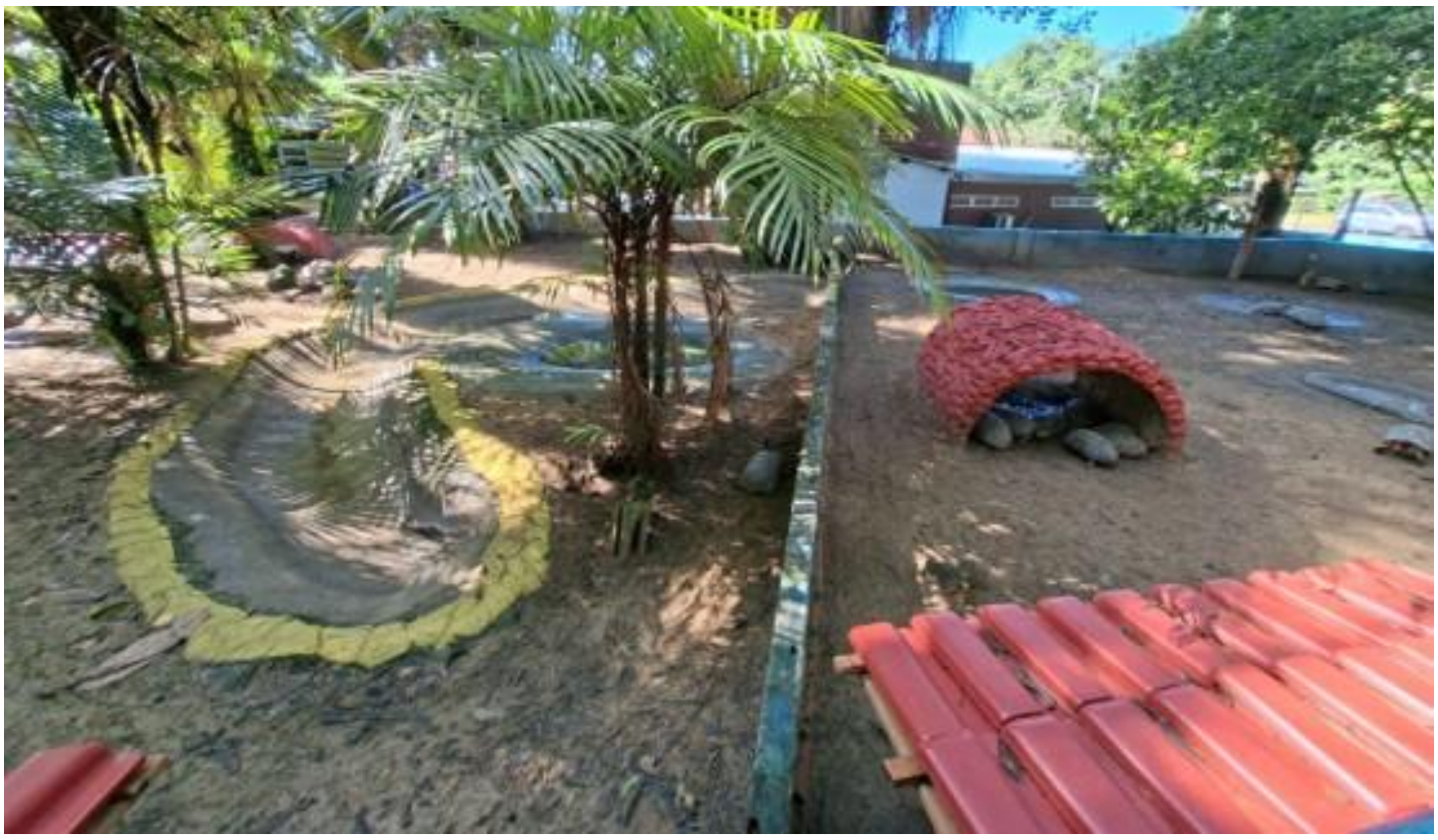

Supplement: Supplementary file 1 [file rbpv-35-3-e013826-suppl01.pdf]
