# Supplementary material for: Survey of endoparasites in Chelonoidis carbonarius and Chelonoidis denticulatus kept at the zoo of the Jungle Warfare Training Center (CIGS), Central Amazon
Source: Rev Bras Parasitol Vet. 2026 Sep 25;35(3):e013826. doi: 10.1590/S1984-29612026041 (PMC13671223; doi:10.1590/S1984-29612026041)

Supplementary 3. Endoparasites are observed in fecal samples from Chelonoidis denticulatus. (A) Microscopic field showing: (i) egg suggestive of Angusticaecum spp., (ii) oocyst of Eimeria spp., (iii) oocyst of Cyclospora spp. (B) egg suggestive of Angusticaecum spp. (C) oocyst of Eimeria spp. (D) oocyst of Cystoisospora spp. (E) Microscopic field showing: (iv) cyst of Cyclospora spp., (v) oocyst of Eimeria spp. (F) oocyst of Cyclospora spp.

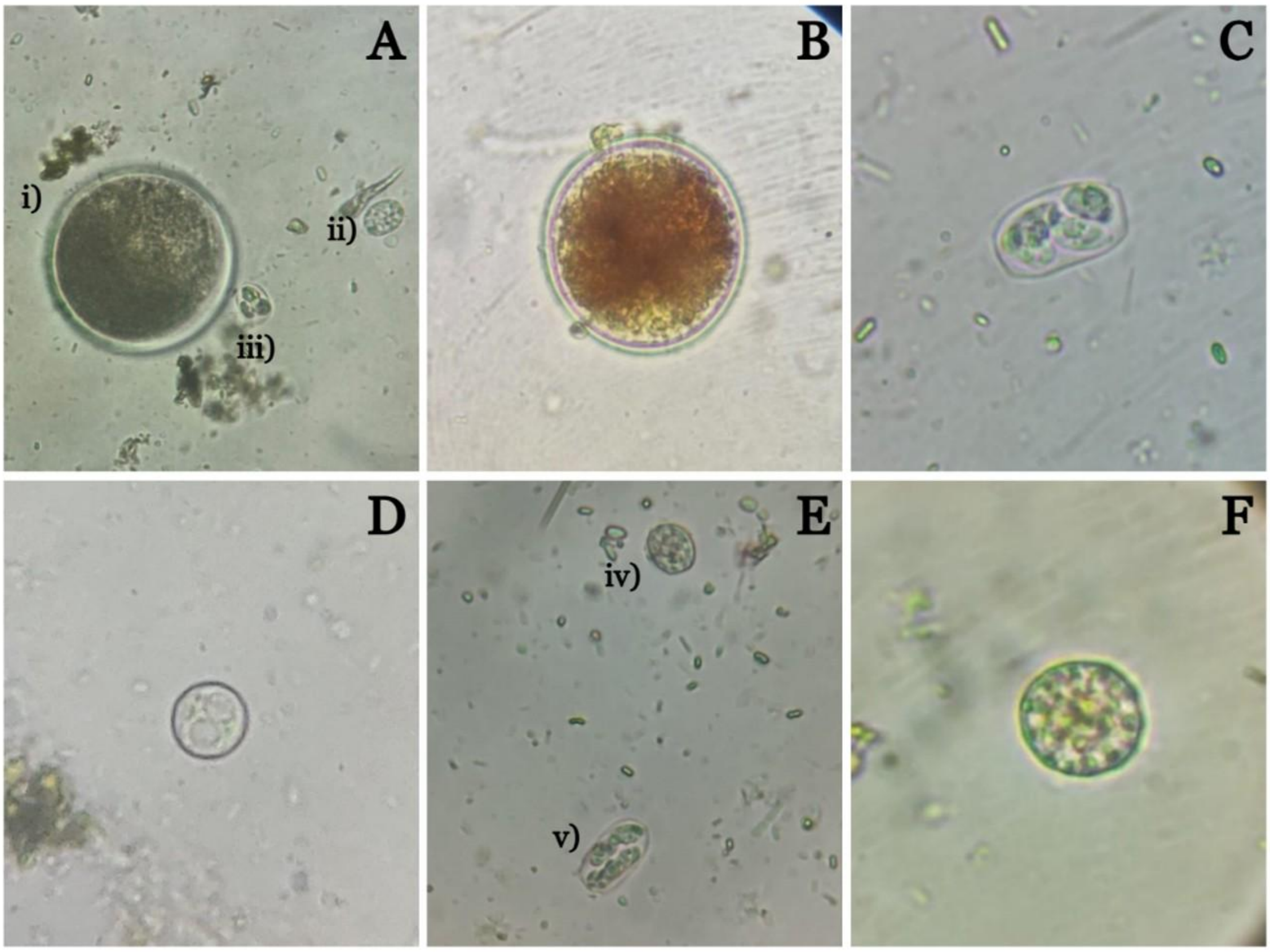

Supplement: Supplementary file 3 [file rbpv-35-3-e013826-suppl03.pdf]
