# Supplementary material for: Survey of endoparasites in Chelonoidis carbonarius and Chelonoidis denticulatus kept at the zoo of the Jungle Warfare Training Center (CIGS), Central Amazon
Source: Rev Bras Parasitol Vet. 2026 Sep 25;35(3):e013826. doi: 10.1590/S1984-29612026041 (PMC13671223; doi:10.1590/S1984-29612026041)

Supplementary 4. Endoparasites observed in fecal samples from Chelonoidis carbonarius. (A) Ciliophora trophozoite (40x magnification). (B) Strongyle type egg (100x magnification).

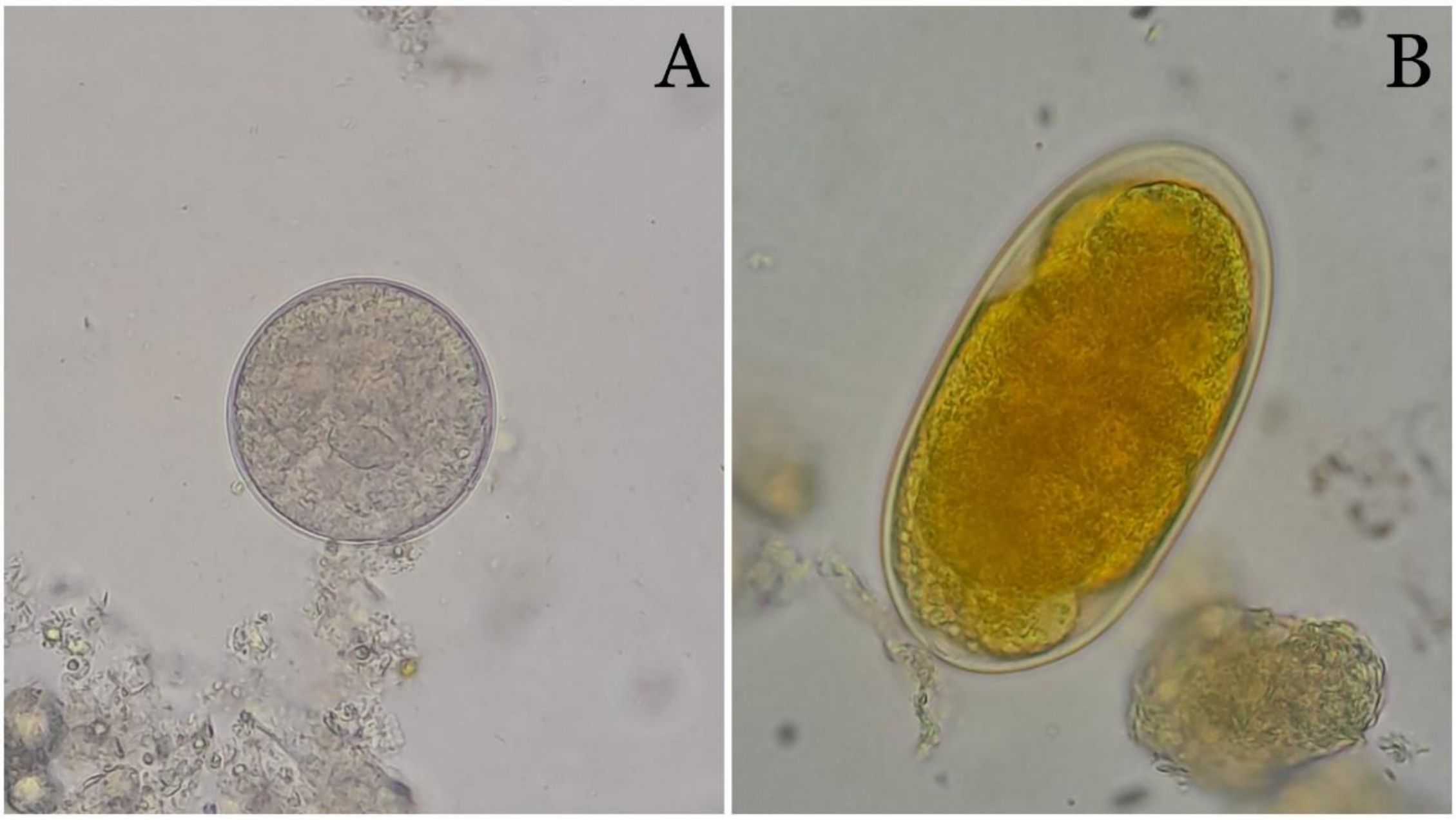

Supplement: Supplementary file 4 [file rbpv-35-3-e013826-suppl04.pdf]
